# Antibiotic Resistance Patterns and Virulence Determinants of Different SCCmec and Pulsotypes of *Staphylococcus Aureus* Isolated from a Major Hospital in Ilam, Iran

**DOI:** 10.2174/1874285801711010211

**Published:** 2017-10-31

**Authors:** Mehdi Abbasi, Majid BaseriSalehi, Nima Bahador, Morovat Taherikalani

**Affiliations:** 1 Department of Microbiology, Shiraz Branch, Islamic Azad University, Shiraz, Iran; 2 Department of Biology, Ilam Branch, Islamic Azad University, Ilam, Iran; 3 Clinical Microbiology Research Center, Ilam University of Medical Sciences, Ilam, Iran

**Keywords:** MRSA, PFGE, SCC*mec* typing, Ilam, Iran, Antibiotic

## Abstract

**Aims & Objectives:**

The aim of this study **is** to evaluate genetic relatedness, antibiotic resistance pattern, and virulence characteristics of different types of *S. aureus* isolated from air, surfaces, staff, and patients in a Public hospital in Ilam.

**Methods & Materials:**

A total of 88 of 140 staphylococci identified as *S. aureus* by conventional and molecular methods were used in this study. Isolate samples were obtained from surfaces, staff, patients, and hospital indoor air. The sampling from staff and surfaces was done through using swab and air by standard pump. Antimicrobial susceptibility testing and presence different resistant and virulence determinants was assessed. Isolates were then typed by pulsed-field gel electrophoresis (PFGE) and SCC*mec* typing methods.

**Results:**

Out of 88isolates, 36 of them (40.9%) were MRSA. Among MRSA isolates, the range of resistance to antibiotic was 0% in vancomycin to 83.3% in gentamycin. The most prevalent resistant genes among gentamicin resistant *S. aureus* were *acc (6')/aph (2”)Ia* and *aph(3”)IIIa*. The most common erythromycin resistant gene was *erm*C. Surprisingly, SCC*mec* types I (30.5%), II (25%)were highly distributed. PFGE analysis showed 33 different pulsotypes.

**Conclusion:**

This study confirms that different isolates of MSSA and MRSA circulate in Ilam which differ in antimicrobial susceptibility, content of resistance, and virulence determinants.

## 1 INTRODUCTION

Nosocomial infections are among important factors inpatient's mortality in Iran and other countries. Hospital environments have been widely identified as the main source of multidrug-resistant bacteria, most importantly MRSA strains. MRSA identification is vital for controlling and eliminating the outbreaks [1-3].

*S. aureus* is a Gram-positive, catalase-positive, non-spore forming facultative anaerobic bacteria. This bacterium can colonize in the nostril's anterior section, vagina, armpit, perineum, skin (especially damaged areas), newborns'umbilicus, and oropharynx [1]. These opportunistic pathogens show various virulence factors such as their adhesion, enzymes, and different toxins. The most important transmission route to patients is through the contaminated hands of healthcare staff working in the hospitals [1, 2]. Methicillin resistant strains of *Staphylococcus aureus*(MRSA) are also commonly resistant to other antibiotics such as erythromycin, beta-lactamases, and cephalosporin and are capable of producing several virulence factors such as exfoliative toxin, hemolysins, and enterotoxins. Due to the increased prevalence of MRSA in Iran, controlling the infection is more important than the past. *S. aureus* has undergone significant changes in antimicrobial susceptibility patterns during the past years according to the geographical area. It is noteworthy that after *Escherichia coli*, it is considered as the second leading cause of nosocomial infections [4]. The resistance to antibiotics among *S. aureus* isolates has increased injuries and mortality rates in hospitalized patients in this city. Nowadays, 30 to 50 percent of *S. aureus* strains have become resistant to methicillin. The presence of *mecA* gene in *S. aureus* genome, normally carried out in cassette chromosome *mec* or SCC*mec,* encodes methicillin resistance ability [3-5].

The present study is implemented in order to evaluate the antibiotic resistance pattern and virulence characteristics of different types of susceptible MRSA using PCR, SCC*mec* typing, and pulsed field gel electrophoresis (PFGE) methods at Mustafa Khomeini Hospital in Ilam in western Iran. The main goal of this study is to evaluate the antibiotic resistance pattern and virulence characteristics of different types of *S. aureus* exclusively at Mustafa Khomeini Hospital in Ilam City in order to understand the epidemiology of these infections.

## 2 MATERIAL AND METHODS

### 2.1 Samples Collection and Primary Identification

A total of 140 isolates were collected from all parts of hospital indoor air, surfaces, staff and patients through using Quick Take 30 Sample Pump, swab and clinical samples at Mustafa Khomeini Hospital in Ilam from July 2014 to January 2015. All isolates were confirmed as *S. aureus* by conventional methods including gram stain, catalase, coagulase, DNase, and mannitol fermentation. Further confirmations were obtained through the use of molecular methods including PCR of *nucA* and *spa* genes. Isolates that were gram-positive, catalase-positive, coagulase-positive, and DNase-positive and also the ones that carried *spa* and *mec*A genes were entered into the study [6, 7].

### 2.2 Antibiotic Susceptibility Test

 Antimicrobial susceptibility patterns were determined by the agar disk diffusion methods according to CLSI guidelines [8]. The tested antibiotics included gentamicin (10μg), erythromycin (15μg), clindamycin (2μg), doxycycline (30μg), minocycline (30μg), ciprofloxacin (5μg), trimethoprim-sulfamethoxazole (5μg), rifampin (5μg), quinupristin-dalfopristin (15μg), and linezolid (30μg) [6-8].

The sensitivity and resistance to methicillin were investigated using cefoxitin (30µg) disk. All MRSAs were further identified by agar dilution of oxacillin according to CLSI guidelines and then confirmed by *mecA* gene detection using PCR after DNA extraction by phenol chloroform based on the past reports [6-8]. Minimum inhibitory concentration (MIC) of vancomycin was also determined by micro-broth dilution. *S. aureus* ATCC25923 and ATCC 29213 were used as the internal control as well [6, 7].

### 2.3 Molecular Detection of Resistant Genes and Virulence Determinants

 Molecular detection of resistant and virulence genes was performed through using PCR amplification after DNA extraction through the use of phenol chloroform (PCI) method(Zhang *et al*., 2005).Specific primers for target genes were conducted using primers sets and cycling conditions that were previously described(Zhang *et al*., 2005).Antibiotic resistant genes (*femA*, *blaZ, ermA, ermB, ermC, msrA, linA, acc(6')/aph(2”), Ia acc(6')/aph(2”)Ie, aph(3”)IIIa, aph(2”)Ib,aph(2”)Ic, aph(2”)Id, and ant(4')Ia* and virulence genes (*tst, hlg, etb, sea, hla, hld, see, seb, sec, sed, hlb, eta, pvl*) of *S. aureus* were used in Multiplex-PCR technique according to the past reports [9-11].

Amplification was carried out in a 25µl volume containing 0.5-1mmol of each primer, 0.75µmolof each dNTP, 1.5mM MgCl2, 1 U TaqDNApolymerase, 2.5 µl of PCR buffer, and 3µl of template. The DNA template was replaced with water and then used as negative control. An initial denaturation at 95 ^◦^C for 5 minutes was followed by 30 cycles of denaturation at 95 ^◦^C for 60 seconds, and then annealed at 54 ^◦^C for 120 seconds, and after that extended at 72 ^◦^C for 120 seconds. Finally, an additional extension was achieved for 5 minutes at 72 ^◦^C^6^.For each PCR product, a10 µl aliquot was electrophoresed on a 1.5% agarose gel for 1.5 h at 100 V, stained for 10minutes in ethidium bromide (0·5µg/ml) and then visualized and photographed under UV illumination with Gel Doc apparatus (BioRad. ChemiDoc XRS+ system).

### 2.4 SCC*mec* Typing

 Multiplex-PCR technique was also used to determine different types of SCC*mec* with 10 specific primers that were designed by Zhang *et al* [9]. Moreover, gene amplification was performed with Multiplex-PCR reaction mixture and designed heat cycle by Zhang *et al*,with a few changes that were optimized by this study [11]. MRSA reference strains including COL (SCC*mec* type I, *ccr*1), XU642 (EMRSA-16, SCC*mec* type II, *ccr*2), WBG525 (EMRSA-1, SCC*mec* type III, *ccr*3), WBG9465 (EMRSA-15, SCC*mec* type IV, *ccr*2), and WIS (SCC*mec* type V, *ccr*C) were used as controls [12].

### 2.5 PFGE

(Pulsed-field gel electrophoresis): Analysis of the genetic similarity between *S*. *aureus* isolates was performed through using PFGE method in accordance with a previously published protocol [13, 14]. Restriction enzyme digestion was performed with 30 U of *SmaI* enzyme in Tango buffer (Thermo Fisher Scientific USA) [13]. Electrophoresis was also conducted in a CHEF-Mapper Bio-Rad Laboratories Unit applying parameters including starting pulse (5s), ending pulse (25s), voltage (6V/cm), and run time (19h). After that, Gel-Compar (Applied Maths, Sint-Martens-Latem, Belgium) was used for cluster analysis using the Dice coefficient and unweighted pair group method with arithmetic mean. Isolates clustered ≥ 75% were considered as the same clone [13, 15].

### 2.6 Statistical Analysis

 The chi-square and Fisher’s exact test were used where appropriate using SPSS (version 20). A two-sided P value of < 0.05 was considered statistically significant.

## 3 RESULTS

### 3.1 Samples Collection and Primary Identification

 A totalof140 gram positive cocci that collect in this period identified as *Staphylococcus spp*., and 88 of them were then more identified as *S. aureus*. *S. aureus* isolate belonged to different samples including surfaces (n=34; 38.9%), staff (23; 26.6%), air (20; 23%) and patients (11;11.9%).

**Fig. (1):**
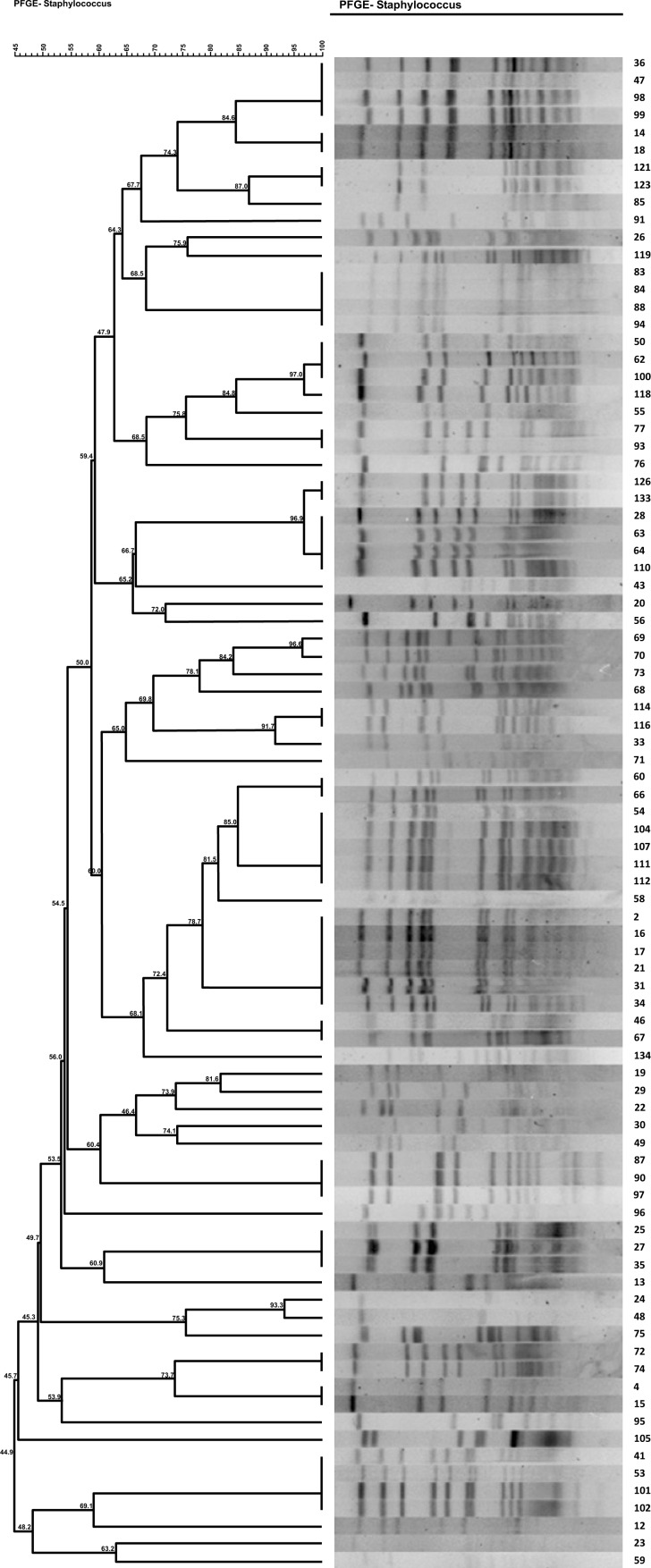
Dendrogram of PFGE clusters of 36 MRSA isolates and their relationship.

From a total of 36 (40.9%) MRSA isolates that were found,26 (72.2%) were isolated from surfaces, 5 (13.8%) from indoor air, 4 (11.1%) from staff and 1(2.7%) from a patient. All the 36 MRSA isolates were both *mecA* positive and cefoxitin resistant. The origin of all 36 MRSA isolates was shown in (Fig. **1**).

### 3.2 Antibiotic Susceptibility

The antimicrobial susceptibility patterns of *S. aureus (*MRAS and MSSA*)* Isolates to various antibiotics are presented in Table (**1**). All *S. aureus* isolates were susceptible to vancomycin, linezolid, and minocycline with resistance rates of 1.1%, 4.5%, and 6.8% that shows good performance against both MRSA and MSSA. As shown in Table (**1**), resistance to antibiotics was more frequent in MRSA than in MSSA.

### 3.3 Frequency of Resistant Genes and Virulence Determinants

 All MRSA isolates harbored *mecA* and *femA* genes. The predominant AME genes were *acc (6')-Ie-aph (2”)Ia* (n=25/40; 62.5%), *aph(3”)-IIIa* (n=12/40; 30%), *acc(6')-Ie-aph(2”)Ie* (n=11/40; 27.5%), and *ant(4')-Ia* (n=6/40; 15) among which co-existence of two genes included 35% (n=14/40) of the isolates. According to Table (**3**), all gentamicin resistant MRSA and MSSA isolates harbored at least one AME gene. However, no other AME gene was detected in the study. The *msrA* gene was highly distributed among macrolide and lincosamide resistant isolates. Besides, *msrA* was found in 46.8% (22/47) of erythromycin, 46.6% of clindamycin, and 36.3% of erythromycin and clindamycin resistant isolates.

It was also found that 21.2% and 19.1% of erythromycin, 26.6% and 33.3% of clindamycin, and 4.2%of clindamycin and erythromycin resistant isolates contained *ermA* and *ermC*.

As shown in Table (**2**), *ermB* and *linA* were found in MSSA but not in MRSA. All clindamycin and also erythromycin and clindamycin co-resistant MRSA and MSSA isolates are carried out at least one resistant gene which was reduced to 66.6% and 76.4% in MRSA and MSSA erythromycin resistant isolates, respectively.

The predominant exotoxins were found to be *sea* (30/88; 34.1%), *tst* (30/88; 34.1%), and *hlg* (113/88; 14.7%). Virulence determinants were revealed to be highly distributed among MSSA isolates. The *eta* exfoliative toxin was also found in 22.2% of MRSA isolates (Table **4**).

### 3.4 SCC*mec* Typing

 Of 36 MRSA isolates, 11 (30.5%) were identified as SCC*mec* type I,9 (25%) as SCC*mec* type II (25%), 8 as SCC*mec* type IV (22.2%), and 3 (8.3%) as type III (8.3%). Five (13.8%) of the isolates were also not identified as any SCC*mec* type. The distribution of major SCC*mec* types according to antibiotic resistance is shown in Dendrogram1 and Table (**5**). As show in the table SCC*mec* type I, II and III were more distributed among the isolates and contain more resistant genes and virulence determinant than SCC*mec* type IV.

### 3.5 PFGE

 (Pulsed-field gel electrophoresis). PFGE results showed 33 different pulsotype patterns. The major pulsotypes among MRSA isolates were 15 (6/33;18.1%), 6 (6/33; 18.1%), and 1(4/33; 12.1%). The distribution of major pulsotypes according to antibiotic resistance was shown in Table (**5**). Pulsotype patterns 15, 6, 8, and 1 had the highest abundance among all MRSA and MSSA isolates. The pulsotype patterns with the lowest abundance included patterns 3, 7, 9, 10, 11, 14, 17, 13, 20, 21, 25, 23, 22, and 30 were singleton. Also, 8 pulsotypes in 11 samples of patients, 17 pulsotypes in 30 isolates of staff, 16 pulsotypes in 24 different isolates of air, and 40 pulsotypes in 16 isolates of surfaces were identified. According to Fig. (**1**) same pulsotypes such pulsotype 15 were identified among different ward.

## 4 DISCUSSION

Nosocomial infections are among the important factors of patient's mortality in Iran and other countries around the world [16, 17].

Bacterial identification and their antibiotic resistance are significant for the prevention and treatment of bacterial infections. Hospital environments have been widely identified as the main source of multidrug-resistant bacteria most importantly MRSA strains [18]. The development of bacterial resistance was linked with antibiotic use and hence selective pressure which was specific for the type of antibiotic and the bacterial species [18]. Antibiotics arguably constitute the most concentrated selective pressure ever brought to bear on S. aureus in its long co-evolutionary history with mankind. The consequences of this selective pressure in conjunction with horizontal and vertical gene transfer are the subject of this review. Given their critical importance as therapeutic agents, the story will focus on resistance to penicillin’s and the structurally related beta-lactam antibiotics [19].

The resistance to antibiotics is one of the main threats to human life in the present century [20]. Bacterial contamination of different parts of hospitals is due to various factors such as physical structure, devices and medical equipment multiplicity, inefficient purification systems, presence of certain diseases, and *etc*. Based on the guidelines of Infection Control Committee of the hospital, hospitalization of patients more than fifteen days in different units of hospital including intensive care units, enhances the risk of nosocomial infections especially by MRSA strains [21]. These clonal complexes were widespread prior to emergence of methicillin resistance indicating that superior epideictic preceded acquisition of drug resistance and that the adaptations and innovations that make clones successful also may favor their adaptation to antibiotic selective pressures [22].

The reasons for the disparity in rates of quinolone resistance between MSSA and MRSA strains are uncertain. One contributing factor is likely antibiotic selective pressure, especially in the hospital setting, resulting in the selection and spread of the more antibiotic-resistant MRSA strains [10].

Several studies have been conducted worldwide on toxic genes of MRSA from various dimensions. In similar clinical studies, the frequency of *sea* gene of MRSA was 58.8% in Gorgan-Iran, 74.4% in Tehran-Iran, 33% in China, and 12% in Germany, while the frequency of *sea* gene in MRSA isolates from various specimens obtained in this study was 25% which is similar to that of the study conducted in Korea [10, 23-26]. Moreover, the frequency of ***sub*** gene has been investigated in many studies. The frequency of this gene in Gorgan, Tehran, China, Canada, Korea, Czech, and Colombia was 61.3%, 73.58%,5%, 15.78%, 5.6%, 3%, and 7%, respectively [27-29]. In this study, the frequency of ***sea*** gene was found to be 8.3%. Another resistance mechanism that was evaluated was macrolide resistance among MRSA isolates. In accordance with an earlier report from Iran [21], resistance rates to erythromycin and clindamycin in this study were discovered to be 50% and 27%, respectively. Also, the resistance rates to erythromycin and clindamycin were 57% and 17%, respectively which were lower than those reported in other studies [30, 31].

Earlier studies have indicated that *ermA* (13.8%) has the most important role in macrolide resistance [16]. However, this study showed *ermC* (11.1%) to be the most prevalent gene among macrolide-resistant MRSA in West of Iran and that *ermB* didn’t play an important role in macrolide-resistant MRSA which is contradictory to the previous reports from Iran [32].

Similar to earlier studies, no *ermB* gene was found among macrolide-resistant MRSA isolates in the current study. In similar studies, the prevalence rate of *ermB* was seen in just a few erythromycin-resistant staphylococci [33]. The results also showed that the resistance to erythromycin was due to the existence of *ermA* among MRSA isolates in Ilam.

The study of antibiotic resistance identified the abundance of several macrolide resistant genes including *ermC*, *ermA, ermC*, *ermB* and *lin*A in this work. However, the frequency of these genes appeared to be lower than that in the previous study by Aktas *et al* [34]. After *ermC* gene with 10.2% abundance, *ermA*, *ermB*, and *linA* genes with respective 5.7%, 4.5%, and 2.3% abundances, were identified as the most common macrolide resistance genes in this study which were lower than the obtained results by Aktas *et al* [34]. Gene *aac (6')-Ie-aph (2”)-Ia* was the most common aminoglycoside resistance gene with 22.7% abundance. Subsequently, the *aph (3”)-IIIa* gene was the second most common one with12.5% abundance. In the current study, as confirmed in many reports [32-34], *acc (6')-Ie-aph (2”)Ia* was the most prevalent AME gene that was encountered in more than 75% of all aminoglycoside-resistant isolates. However, reports from Kuwait and Japan indicated that the prevalence of *ant (4')-Ia* gene is much higher than that of the other three AME genes found in the present study). The most frequently encountered AME in staphylococci is *acc(6')-Ie-aph (2”)Ia* which deactivates the wide range of medically important aminoglycosides. Moreover, 46.6% of MRSA isolates in this study were also positive for *acc(6')-Ie-aph (2”)Ia*. The majority of these isolates (95%) was resistant to all tested aminoglycoside antibiotics [35]. The second most detected AME gene in this study is *aph(3”)-IIIa* and the enzyme encoded by it confers resistance to amikacin, but not gentamicin. The rate for *ant(4')-Ia* in this study (10%) was lower than those reported from Kuwait (87%) and European countries (53%) [36]. However, the rates of *aph(3”)-IIIa* and *ant(4')-Ia* differ greatly among different countries. Except for 7 aminoglycoside-resistant strains, the rest contained AME genes in this study revealing the importance of these enzymes in the modification of aminoglycoside antibiotics among staphylococci in Ilam.

Compared to other studies, a much higher frequency of *tst* gene was found in this study which raised a significant concern for its sanitation and hygienic practices [36]. Immediate regulatory action should be taken to reduce its nosocomial infection rate and prevent the spread of *S. aureus* among patients and staff [35, 36]. Given that most isolated strains of this gene existed on the surfaces, regulatory actions of authorities are necessary to be taken in order to control the environmental health in hospitals and prevent the spread of these strains among patients and staff. The frequency rates of *eta* gene reported from Germany, Czech Republic, Turkey, and Colombia were 2%, 10%, 19.2%, and 3%, respectively [36-38] while it was equal to 22.2%in this study. The frequency of *etb* gene in a study carried out in Turkey was 9.2%. In Colombia, however, no *etb* gene was detected among 30 MRSA [38]. The frequency of the *etb* in this study was also obtained 6.8%.

The most predominant hemolysin gene among MRSA and MSSA isolates was *hlg* gene. However, the frequencies obtained in this study (84.24% abundance) were lower than those obtained in the study by Kim *et al* (93.15% abundance) [39]. The frequency of *hla* gene, in a study by Hoseini Alfatemi *et al*. in Shiraz was reported to be 93.1% [11].In this study however, *hla* gene with a frequency of 93.15% was the most abundant one. According to studies conducted in other parts of the world, we can conclude that this gene is comparatively much more frequent in MRSA isolates [11].

In the present study, four types of SCC*mec* were identified among 36 strains of MRSA. However, SCC*mec* type V was not detected in isolated strains. The strains with the highest abundance were SCC*mec* type I with 30.5% and then types II, IV, and III with 25.0%, 22.2%, and 8.3%, respectively.

Similar results were reported in Spain by Pereze *et al* (Shukla *et al*., 2010). (1998-2000) and 2.9% of 375 MRSA strains were not typed in this study. In the reports of Chung *et al*. (2004) and Zhang *et al*., among all types, type IV has the highest abundance (50%). Kim *et al*. (2006) in South Korea reported the abundance of type IV to be 68.8% which is higher than the obtained results in this study [40].

In another study, it has been reported [41] that type III with 73% abundance was the most detected SCC*mec* type [42].

In this study, the most abundant SCC*mec* type was related to CCU with 36.3%. All these strains were isolated from shock devices, monitors, and patient’s urine. Accordingly, various hospital surfaces and patients contributed to circulation of these strains. Therefore, proper routines sanitation of surfaces should be immediately implemented [40, 42].

The highest abundance of *SCCmec* type II and IV was seen in the CCU and the lowest abundance was observed in obstetrics surgery and emergency units. The presence of these similar strain types in different units of a hospital strongly suggests the contamination and circulation of these strains between surfaces and patients [42].

According to the results obtained in this study, SCC*mec* type II had the highest rate (62.5%, 8/5) among various SCC*mec* types. Strains isolated from staff also had the highest SCC*mec* type III (60%, 5/3).Similarly, the most frequent strain in air and surfaces was SCC*mec* type IV (50%, 2/1).It has been already shown that SCC*mec* types I to III are frequently isolated from hospital-acquired MRSA and often the *mecA* gene is one of the additional genes playing a role in resistance to several beta-lactam antibiotics [42]. Several reasons for gaining additional resistant genes by these isolates have been reported in medical centers that could explain the high antibiotic resistance of SCC*mec* type I-III isolates in this study. Moreover, the significant antibiotic susceptibility pattern of certain SCC*mec* type isolates obtained in this study can prompt us to consider these antibiotics as an option for the treatment of infection caused by such isolates. Most type IV isolates in this study were susceptible to the majority of the tested antibiotics. However, similar to some studies, a few isolates containing type IV cassette were also multidrug-resistant in this study. This suggests that since type IV isolates are often exposed to antibiotics, they could gain antibiotic resistant genes other than *mecA* gene to survive in medical centers [7]. The frequency of SCC*mec* type I isolates in the present study shows the emergence of this type in the studied medical centers. As was previously mentioned in the results section, due to the significant clinical sources and the antimicrobial susceptibility patterns of different SCC*mec* type isolates, optimizing institutional infection control policies for preventing MRSA transmission among hospitalized patients must be considered [7, 12]. Several molecular typing methods such as DNA fingerprint by PFGE and MLST are best known for typing MRSA isolates. As a gold standard typing technique, it has been used for its reliability, discriminatory, and reproducibility in regional studies [12, 40, 42, 43]. It has also been stated that PFGE of *S. aureus* is the most discriminant genotyping tool and a good method to resolve clonal relationships. Javidnia *et al* reported 4 different types of *S. aureus* isolated from intensive care units (ICU) of three different hospitals in Tehran. In their study, PFGE typing method revealed differences in pulsotypes similar to the results obtained in this study [40]. Moreover, in another similar study conducted by Emaneini *et al* using PFGE in Tehran hospitals, different pulsotypes were isolated with7 types being detected in only one isolated *S. aureus* [18].

Rahimi *et al* also reported that diverse pulsotypes consisting of 13 common types and 18 single types along with seven common PFGE types were found among the MRSA strains [33]. Lastly, in a study by Aris de Sousa in Portugal, 271 MRSA isolates were typed by the PFGE typing method and 13 types and 63 subtypes were detected, 5 types of which were typed at least in ten isolates [44]. The results of these studies showed that the origin of all isolates was not identical and there was no significant relationship between them which are consistent with the results obtained in the present study. Moreover, the results of this study indicated that the abundance of pulsotype patterns was different in isolated strains of different samples taken from the hospital. The obtained results also showed that there was no significant correlation between pulsotype patterns of isolated strains in patients and isolated strains of staff, hospital-air, and hospital surfaces. Moreover, different isolates of *S. aureus* were not of the same clonal origin(Aires de Sousa de Lencastre. 2004). Pulsotype patterns of *S. aureus* showed that 75% of pulsotype patterns was different. However, different pulsotypes were observed in this study with no significant relationship between them. Based on the obtained results, pulsotypes1, 2, 11, and 16 were observed in all the understudy parts of the hospital which expressed a relationship between *S. aureus* isolates in different populations. These findings reflect the urgent need to control and eliminate *S. aureus* infection. Accordingly, rigorous regulations and policies should be adopted for sanitation or disinfection programs. Proper indoor air ventilation is also needed and minimal antibiotic therapy should be administered among patients [6, 7, 18, 44].

Due to their high discriminatory power, PFGE techniques are considered as a useful tool for determining the prevalence of *S. aureus* infections particularly in local hospitals. In the past, it was difficult to analyze and compare the obtained information of PFGE from different hospitals and evaluation of the obtained results for each hospital was only used for itself.

## CONCLUSION

 The obtained results in this study, like those of other studies conducted in Iran and other parts of the world, show high prevalence of *S. aureus* isolates in various units of hospitals, equipment, staff and patients. The PFGE typing technique is used as a standard molecular typing method to determine the relationship between isolated *S. aureus* of different parts in the hospital.

The obtained results indicated that the highest and lowest abundance of *S. aureus* was found on hospital surfaces and in patients, respectively. These findings confirm two points. First, surfaces are the main sources of contamination and infection in the hospitals as they transfer the center of *S. aureus* isolates into different parts of a hospital. Second, in Mostafa Khomeini Hospital in Ilam, it was more likely for bacteria to be transferred from the hospital environment to patients than the other way around. Different pulsotypes were also identified in this study with some having greater abundance than the others which describe the possibility of a common genetic origin of the isolates. In order to improve and complete the treatment and reduce the incidence of MRSA and also to limit the spread resistance and accurate diagnosis, the following suggestions are offered. Antibiotic resistance and virulence factors should be annually surveyed and clone resistance should be identified. Moreover, hospital entries and exits should be controlled. We also found that the hospital staff and surfaces have very important role in the transmission of infection into the hospitals and can be a way to transfer *S. aureus* strains among different wards of hospitals.

## FUNDING

No funding.

## Figures and Tables

**Table 1:** The antimicrobial susceptibility patterns of *S. aureus* isolates.

Antibiotic	MRSA n=36			MSSA n=52			Total n=88		
	No. (%)			No. (%)			No. (%)		
	R I S			R I S			R I S		
Gentamicin	30(83.3)	1(2.7)	5(13.8)	10(19.2)	5(9.6)	37(71.1)	40(45)	6(6.8)	42(47.7)
Erythromycin	30(83.3)	0(0)	6(16.6)	17(32.6)	2(3.8)	33(63.4)	47(53)	2(2.2)	39(44.3)
Doxycycline	12(33.3)	6(16.6)	18(50)	4(7.6)	7(13.4)	41(78.8)	16(18)	13(14.7)	59(67)
Minocycline	5(13.8%)	6(16.6%)	25(69.4%)	1(1.9%)	2(3.8)	49(94.2)	6(6.8)	8(9)	74(84)
Ciprofloxacin	11(30.5)	6(16.6)	19(52.7)	1(1.9)	2(3.8)	49(94.2)	12(13.3)	8(9)	68(77)
Clindamycin	19(52.7)	3(8.3)	14(38.8)	7(13.4)	3(5.7)	42(80.7)	26(29.5)	6(6.8)	56(63)
Trimethoprim Sulfamethoxazole	14(38.8)	0(0)	22(61.1)	3(5.7)	0(0)	49(94.2)	17(19)	0(0)	71(80)
Rifampin	13(36.1)	0(0)	23(63.8)	0(0)	0(0)	52(100)	13(14.7)	0(0)	75(85)
Quinupristindalfopristin	1(2.7)	0(0)	35(97.2)	0(0)	1(1.9)	51(98)	1(1.1)	1(1.1)	86(97)
Linezolid	1(2.7)	0(0)	35(97.2)	3(5.7)	0(0)	49(94.2)	4(4.5)	0(0)	84(95)

R=Resistant, S=Sensitive, I=Intermediate

**Table 2:** Distribution of resistant gene among erythromycin and clindamycin resistant *S. aureus* isolates.

Genes	Erythromycin n=47		Clindamycin n=15		Erythromycin & Clindamycin n=11	
	MRSA n=30	MSSA n=17	MRSA n=9	MSSA n=6	MRSA n=7	MSSA n=4
	No. (%)	No. (%)	No. (%)	No. (%)	No. (%)	No. (%)
*erm A*	5(16.6)	4(23.5)	2(22.2)	2(66.6)	2(28.5)	1(25)
*erm C*	4(13.3)	1(5.8)	2(22.2)	1(16.6)	1(4.2)	1(25)
*erm B*	0(0)	3(17.6)	0(0)	1(16.6)	0(0)	1(25)
*lin A*	0(0)	2(11.7)	0(0)	1(16.6)	1(0)	0(2.1)
*erm A + erm C*	1(3.3)	0(0)	0(0)	0(0)	0(0)	0(0)
*msr A*	7(23.3)	10(58.8)	3(33.3)	1(16.6)	2(8.5)	1(25)
*msr A+ erm C*	3(10)	0(0)	2(22.2)	0(0)	1(4.2)	0(0)
*msr A+ lin A*	0(0)	2(11.7)	0(0)	1(16.6)	0(0)	0(0)
*Total*	20(66.6)	13(76.4)	9(100)	7(100)	7(100)	4(100)

**Table 3:** Distribution of virulence genes among MRSA and MSSA isolate

Gene	MRSA n=36	MSSA n=52	Total n=88
	No. (%)	No. (%)	No. (%)
*hla* *hlb* *hlg* *hld*	0 (0)	1(1.9)	1 (1.1)
	0 (0)	0(0)	0 (0)
	4(8.3)	9(17.3)	13 (14.7)
	0 (0)	0(0)	0 (0)
*sea* *seb* *sec* *sed* ***s****ee*	9(25)	21(40.3)	30 (34.1)
	0 (0)	0(0)	0 (0)
	0 (0)	0(0)	0 (0)
	0 (0)	0(0)	0 (0)
	3(8.3)	6(11.5)	9 (10.2)
*eta*	8(22.2)	1(1.9)	9 (10.2)
*etb*	1(2.7)	5(9.6)	6 (6.8)
***t****st*	11(30.5)	19(34.6)	30 (34.1)

**Table 4:** Distribution of virulence genes among MRSA and MSSA isolate.

Gene	MRSA n=36	MSSA n=52	Total n=88
	No. (%)	No. (%)	No. (%)
*hla* *hlb* *hlg* *hld*	0 (0)	1(1.9)	1 (1.1)
	0 (0)	0(0)	0 (0)
	4(8.3)	9(17.3)	13 (14.7)
	0 (0)	0(0)	0 (0)
*sea* *seb* *sec* *sed* ***s****ee*	9(25)	21(40.3)	30 (34.1)
	0 (0)	0(0)	0 (0)
	0 (0)	0(0)	0 (0)
	0 (0)	0(0)	0 (0)
	3(8.3)	6(11.5)	9 (10.2)
*eta*	8(22.2)	1(1.9)	9 (10.2)
*etb*	1(2.7)	5(9.6)	6 (6.8)
***t****st*	11(30.5)	19(34.6)	30 (34.1)

**Table 5:** Distribution of major SCCmec and pulsotypes among MRSA isolates according to antibiotic resistant phenotype.

Antibiotic Resistant Phenotypes	No. (%)	Major SCCmec Types No. (%)					Major PulsotypesNo. (%)				
		I	II	III	IV	N	1	6	12	15	31
**C**efoxitinC	36(100)	9(25)	10(27)	7(19.4)	6(16.6)	4(11.1)	4(12.1)	6(18.1)	1(3)	6(18.1)	2(6)
Gentamicin	30(83.3)	9(25)	7(19.4)	7(19.4)	5(13.8)	2(5.5)	3(9)	6(18.1)	1(3)	5(15.1)	1(3)
Erythromycin	30(83.3)	8(22.2)	7(19.4)	6(16.6)	5(13.8)	4(11.1)	3(9)	6(18.1)	0(0)	5(15.1)	0(0)
Doxycycline	12(33.3)	3(8.3)	2(5.5)	4(11.1)	1(2.7)	2(5.5)	1(3)	2(6)	0(0)	3(9)	0(0)
Minocycline	5(13.8)	3(8.3)	1(2.7)	1(2.7)	0(0)	0(0)	1(3)	2(6)	0(0)	2(6)	0(0)
Ciprofloxacin	11(30.5)	5(13.8)	3(8.3)	2(5.5)	1(2.7)	0(0)	3(9)	2(6)	1(3)	1(3)	0(0)
Clindamycin	19(52.7)	5(13.8)	3(8.3)	6(16.6)	3(8.3)	2(5.5)	3(9)	3(9)	1(3)	2(6)	1(3)
Trimethoprim	14(38.8)	3(8.3)	2(5.5)	3(8.3)	4(11.1)	2(5.5)	2(6)	2(6)	1(3)	2(6)	1(3)
Rifampin	13(36.1)	6(16.6)	2(5.5)	4(11.1)	0(0)	1(2.7)	1(3)	2(6)	1(3)	1(3)	1(3)
Quinupristindalfopristin	1(2.7)	0(0)	0()	0(0)	0(0)	1(2.7)	0(0)	0(0)	0(0)	1(3)	0(0)
Linezolid	1(2.7)	0(0)	0()	1(2.7)	0(0)	0(0)	0(0)	0(0)	0(0)	0(0)	1(3)

**Table 6:** Phenotypic and genotypic features of 36 MRSA isolates in this study.

Sample No	**Ward**	**Origin**	**Resistance** **Phenotype**	**Pulsotypes**	**SCC *mec***	**Resistant Genes**	**Virulence Genes**
55	Women, internal	Bed	FOX, GM,E, D,MI, CIP, RA	6	I	*blaZ, aph(6)-IIIa*	*sea, tst*
62	Women, surgery	Computer	FOX, GM,E, CIP,DA, TMP, RA	6	I	*blaZ*	*tst*
77	NICU	Air	FOX, GM,E	6	II	*blaZ, acc(6')-Ie-aph(2”)Ia, ermA*	*-*
93	Men, internal	Tralee	FOX, GM,E, DA, TMP	6	IV	*aac(6)Ie-aph(2)-Ie, blaZ, acc(6')-Ie-aph(2”)Ia, aph(6)-IIIa, ermC, msrA*	*see*
100	ICU	Sink	FOX, GM,E, DA	6	I	*blaZ, acc(6')-Ie-aph(2”)Ia, ermA*	*etb, sea, see,*
118	CCU	Bed	FOX, GM,E,MI, CIP, DA, RA	6	III	*blaZ, acc(6')-Ie-aph(2”)Ia*	*tst*
31	CCU	Station	FOX, GM,E, D,MI, RA	15	II	*blaZ*	*eta*
34	Women, surgery	Mobile staff	FOX	15	N	*blaZ*	*eta*
54	ICU	IPhone	FOX, GM,E, D, CIP,DA, TMP	15	III	*blaZ*	*sea, tst .eta*
104	Men, Post CCU	Air	FOX, GM,E,MI	15	I	*blaZ, linA, msrA*	*-*
111	Women, internal	Station	FOX, GM,E	15	IV	*blaZ, ermB*	*tst*
14	CCU	Ventilator	FOX, GM,E, CIP, DA, RA	1	I	*aac(6)Ie-aph(2)-Ie, blaZ, acc(6')-Ie-aph(2”)Ia, aph(6)-IIIa, ermA*	*see, tst, sea*
36	CCU	Air	FOX, GM	1	III	*blaZ,, ermA, ermC*	*tst, eta*
47	CCU	Ventilator	FOX,E, CIP, DA, TMP	1	N	*blaZ, acc(6')-Ie-aph(2”)Ia, aph(6)-IIIa, msrA*	*sea .eta*
99	NICU	Incubator	FOX, GM,E,MI, CIP, DA, TMP	1	II	*blaZ, acc(6')-Ie-aph(2”)Ia*	*see, hlg, sea*
25	CCU	Staff=Men	FOX, GM,E, CIP, DA, RA	24	N	*blaZ, acc(6')-Ie-aph(2”)Ia*	*hlb*
27	CCU	Monitor	FOX,	24	I	*blaZ*	*tst, hlg*
**Continue Table 6. Phenotypic and genotypic features of MRSA isolates in this study.**							
**Sample No**	**Ward**	**Origin**	**Resistance** **phenotype**	**Pulsotypes**	**SCC *mec***	**Resistant Genes**	**Virulence Genes**
35	CCU	Air	FOX, E, DA, RA	24	II	*blaZ*	*tst, hlg, eta*
53	NICU	Station	FOX	31	II	*blaZ*	*tst, seb, sea*
102	Emergency department	Ventilator	FOX, GM,E, DA, TMP, RA, LNZ	31	III	*blaZ*	*-*
24	CCU	Staff=Men	FOX, GM,E, CIP, DA, RA	26	II	*blaZ, acc(6')-Ie-aph(2”)Ia*	*tst, hlg*
75	Operating room	Medicine cabinet	FOX, GM,E	26	I	*blaZ*	*hlg, tst*
4	CCU	Staff=Men	FOX, GM,E	28	II	*blaZ, acc(6')-Ie-aph(2”)Ia, ant (4')-Ia, ermC, msrA*	*tst, hlg, sea*
20	Men, Post CCU	Staff=Men	FOX, GM,	10	IV	*blaZ*	*tst*
28	NICU	Medicine cabinet	FOX, GM,E, CIP, DA, RA	8	II	*blaZ, ant (4')-Ia, msrA*	*sea*
29	Women, surgery	Bed	FOX, GM,E, D, TMP	18	N	*blaZ, acc(6')-Ie-aph(2”)Ia,, ermA*	*tst*
30	NICU	Station	FOX, GM,E, D, TMP	20	I	*blaZ, acc(6')-Ie-aph(2”)Ia, aph(6)-IIIa*	*-*
43	ICU	patient=BC	FOX, GM,E, DA, RA	9	IV	*blaZ, ant (4')-Ia*	*tst, eta*
43	ICU	patient=BC	FOX, GM,E, DA, RA	9	IV	*blaZ, ant (4')-Ia*	*tst, eta*
56	Women, internal	Refrigerator	FOX, GM,E,DA, TMP	11	IV	*blaZ*	*sea*
68	Men, internal	Refrigerator	FOX, GM	12	IV	*blaZ*	*sea, tst*
72	NICU	Station	FOX, GM,E, DA	27	III	*blaZ*	*tst, hlg*
83	Men, Post CCU	Air	FOX,E, TMP	5	II	*blaZ, ermA*	*-*
90	ICU	Cover records	FOX, GM,E, D, CIP, DA, RA	22	III	*blaZ*	*-*
91	Women Surgery	Floor	FOX, GM,E, CIP, DA, TMP, RA	3	I	*aac(6)Ie-aph(2)-Ie, blaZ, acc(6')-Ie-aph(2”)Ia, ermC, msrA*	*sea*
96	CCU	Tralee	FOX, GM,E, D, TMP	23	III	*blaZ*	*see*
